# Therapeutic effects of faricimab on aflibercept-refractory age-related macular degeneration

**DOI:** 10.1038/s41598-023-48190-6

**Published:** 2023-11-30

**Authors:** Ryosuke Tamiya, Masayuki Hata, Asako Tanaka, Memiri Tsuchikawa, Naoko Ueda-Arakawa, Hiroshi Tamura, Manabu Miyata, Ayako Takahashi, Ai Kido, Yuki Muraoka, Masahiro Miyake, Sotaro Ooto, Akitaka Tsujikawa

**Affiliations:** Department of Ophthalmology and Visual Sciences, Kyoto University Graduate School of Medicine, Sakyo-Ku, Kyoto, 606-8507 Japan

**Keywords:** Macular degeneration, Molecular medicine

## Abstract

Though vascular endothelial growth factors (VEGF) and other proangiogenic factors, such as angiopoietins (Ang), may be involved in the development of neovascular age-related macular degeneration (nvAMD), only drugs that inhibit the VEGF family are available for the treatment. The newly approved anti-VEGF drug faricimab, which also inhibits Ang-2, is expected to be effective in patients with AMD refractory to conventional anti-VEGF drugs. Therefore, we prospectively investigated the efficacy of faricimab in the treatment of aflibercept-refractory nvAMD. Patients with nvAMD who had been treated with aflibercept in the last year and required bimonthly injections were recruited. 25 eyes showed persistent exudative changes immediately before the faricimab injection (baseline). In these 25 eyes, switching to faricimab did not change visual acuity or central retinal thickness 2 months after the injection; however, 56% of eyes showed reduction or complete absorption of fluid. Notably, 25% of the eyes that showed dry macula at month 2 had no fluid recurrence for up to 4 months. These results indicate that faricimab could benefit some patients with aflibercept-refractory nvAMD.

## Introduction

Age-related macular degeneration (AMD), specifically its late-stage neovascular form (nvAMD), is a significant pathological condition that contributes to vision loss in the aging population^1^. In nvAMD, the pathological neovascularization of choroidal blood vessels beneath the macula or intraretinal neovascularization, termed macular neovascularization (MNV), invades the subretinal pigment epithelium (sub-RPE) space and further into the subretina, destroying photoreceptors within the macula. This pathological condition is associated with numerous factors including vascular endothelial growth factor (VEGF). However, despite the numerous VEGF inhibitors used for the treatment of nvAMD^2–4^, a substantial proportion of patients exhibit resistance or are refractory to these interventions, necessitating alternative or supplementary treatment strategies^5,6^.

Recent advancements in the understanding of the etiology and pathogenesis of nvAMD have highlighted the crucial role of angiopoietins (Ang) such as angiopoietin-2 (Ang-2) in the development of this condition^7^. Although Ang-2 inhibition has long been postulated as a potential therapeutic strategy, the field has been limited by the absence of drugs that simultaneously target both VEGF and Ang. The emergence of faricimab, a novel dual-action drug that inhibits both VEGF-A and Ang-2, has provided a promising approach for the management of nvAMD^8,9^. Pivotal phase 3 TENAYA and LUCERNE studies showed that faricimab is effective in treating treatment-naïve nvAMD^10,11^. However, its therapeutic potential and efficacy in patient’s refractory to conventional anti-VEGF drugs remains unexplored.

This study aimed to fill this knowledge gap by prospectively investigating the efficacy of faricimab in patients with aflibercept-refractory nvAMD. By examining the effects of faricimab in patients with persistent exudative changes despite prior aflibercept treatment, this study may provide invaluable insights into its potential as an effective treatment for nvAMD.

## Methods

### Study design and setting

This prospective, nonrandomized, observational study was conducted in an institutional setting. The study design was approved by the Institutional Review Board of Kyoto University Graduate School of Medicine (R0532), and all study procedures adhered to the tenets of the Declaration of Helsinki. Each patient provided written informed consent to participate in the study.

### Patients and study population

Participants were recruited from the Macular Service, Department of Ophthalmology, Kyoto University Hospital, between June 2022 and October 2022. The inclusion criteria were: age older than 50 years, axial length less than 26.5 mm, the presence of nvAMD, previous treatment with anti-VEGF drugs (aflibercept, ranibizumab, brolucizumab, and pegaptanib) and had received aflibercept injections within the past 3 months and then judged as aflibercept-refractory cases, and willingness to participate in the study. The judgement of aflibercept refractoriness was based on the following: those who exhibited persistent exudative changes despite bimonthly injections of aflibercept. The exclusion criteria were: any previous treatment for MNV; presence of other retinal diseases, such as angioid streaks, vitelliform macular dystrophy, and retinal vein or artery occlusion; patients with a chronic course of AMD, as indicated by disease history and/or massive fibrotic lesions; and patients that dropped out.

### Intervention and observation procedure

All patients received a single dose of faricimab (6.0 mg) and were observed without additional doses for up to 2 months (no loading phase). Clinical data were collected at baseline and 2 months after the faricimab injection. Patients underwent comprehensive examinations including BCVA measurement with Landolt C-charts (Takagi Seiko, Nakano, Japan) in a standard way^12^, axial length measurement (IOL Master, Carl Zeiss Meditec, Dublin, California, USA), fundus photography, spectral-domain optical coherence tomography (SD-OCT; Spectralis; Heidelberg Engineering, Heidelberg, Germany), fluorescein angiography (FA) and indocyanine green angiography (IGA), and fundus autofluorescence imaging at baseline (HRA2; Heidelberg Engineering). Fundus photography, SD-OCT, angiography, and fundus autofluorescence imaging were performed with sufficient pupillary dilation. Visual acuity measurements and SD-OCT were also performed at 4 months, and the results at baseline and 4 months were analyzed. The SD-OCT images were obtained using Spectralis (Spectralis Family Acquisition Module, version 5.7.5.0) and Heidelberg Eye Explorer (version 1.8.6.0; Heidelberg Engineering). The eye-tracking system of the device was used to detect and maintain the correct scanning positions. Thirty horizontal and vertical scans through the fovea were recorded using normal and enhanced depth imaging modes, with an average of 100 scans^13^. Thirteen raster scans covering a 20 × 30-degree oblong rectangle were performed, with an average of 50 scans per session.

We measured central retinal thickness (CRT), which was defined as the distance between the vitreoretinal surface and the inner surface of the RPE. Subfoveal choroidal thickness (SFCT) was measured using enhanced depth imaging scans as the length between the outer border of the Bruch’s membrane and the chorioscleral interface. CRT and SFCT were measured in horizontal and vertical scans and averaged. pigment epithelium detachment (PED) height was defined as the distance between the outer border of the RPE and the inner border of Bruch’s membrane. Graders reviewed all the B-scans to determine and measure the maximum PED height in the raster scans. The presence of fluid accumulation was determined using SD-OCT at baseline and at month 2. Dry macula was defined as complete resolution of intraretinal fluid (IRF) and/or subretinal fluid (SRF) detected on SD-OCT raster scans. The persistence of only PED was considered to indicate a dry macula.

### Definition of subtypes of age-related macular degeneration and pachychoroid neovasculopathy

We judged the presence of polypoidal lesions by using IGA at baseline in each case. Polypoidal choroidal vasculopathy (PCV) was diagnosed on the basis of characteristic polypoidal lesions at the border of the branching choroidal vascular networks. Those with signs of retinochoroidal anastomosis were diagnosed with retinal angiomatous proliferation (RAP), whereas others were diagnosed with typical AMD (tAMD).

In this study, pachychoroid neovasculopathy (PNV) was diagnosed according to the following criteria^14,15^: (1) MNV in either eye; (2) clinical features of the pachychoroid phenotype^16^, such as reduced fundus tesselation on fundus photographs, choroidal vascular hyperpermeability (CVH) on IA images, and dilated choroidal vessels on SD-OCT and IA images. Dilated choroidal vessels were defined as the dilated outer choroidal vessels with attenuation and thinning of the choriocapillaris on OCT^16^. In IA, dilated choroidal vessels extend from one or more vortex veins^17^. (3) No drusen or only non-extensive hard drusen in both eyes (Age-Related Eye Disease Study level 1, no AMD^18^). Diagnoses were made by two ophthalmologists, and in case of discrepancy, a senior retinal specialist determined the final diagnosis.

### Outcome measures

The main outcome measure was change in visual acuity. Secondary outcome measures included subgroup comparisons between patients with and without PNV and those with AMD subtypes.

### Statistical analysis

Statistical analysis was performed using SPSS version 21 (IBM Japan, Tokyo, Japan). A P value less than 0.05 was considered statistically significant. Visual acuity was measured using Landolt C-charts and converted to logarithm of the minimum angle of resolution (logMAR) values for statistical analysis. The unpaired *t*-test or Fisher’s exact test was used to compare baseline characteristics between the tAMD and PCV groups and between the PNV and non-PNV groups. A paired *t*-test was used to compare best-corrected visual acuity (BCVA), CRT, SFCT, and PED height between baseline and 2 months.

## Results

### Study population and baseline characteristics

This study included 25 eyes of 25 patients with nvAMD (19 men and 6 women) who met the inclusion and exclusion criteria. There were 13 eyes with tAMD, 11 with PCV, and one with RAP. Baseline characteristics and clinical data are shown in Table 1. The mean age of the cohort was 77.2 ± 9.1 years. The mean logMAR BCVA at baseline was 0.21 ± 0.18.

**Table 1:** Baseline demographic and clinical characteristics of patients with neovascular age-related macular degeneration.

	Total eyes (n = 25)
Age (y)	77.2 ± 9.1
Sex (male/female)	19/6
Axial length (mm)	23.92 ± 1.02
Lens status (phakia/IOL)	7/18
Subtype (tAMD/PCV/RAP)	13/11/1
Number of previous injections before the switching	
Total	25.3 ± 21.5
Aflibercept	23.6 ± 19.9
Ranibizumab	1.2 ± 3.3
Brolucizumab	0.3 ± 1.0
Pegaptanib	0.1 ± 0.4
Duration since the first treatment (years)	6.0 ± 5.3 (1–20)
Duration since the last injection (aflibercept, wks)	8.1 ± 1.9
Visual acuity (logMAR)	0.21 ± 0.18
Greatest linear dimension (μm)	4639.3 ± 2414.5
Fluid	
SRF (+ / −)	22/3
IRF (+ / −)	8/17
CRT (μm)	193.0 ± 108.6
Subfoveal CT (μm)	174.4 ± 49.7
Maximum PED height (μm)	286.7 ± 160.3

*IOL* intraocular lens; *tAMD* typical age-related macular degeneration; *PCV* polypoidal choroidal vasculopathy; *RAP* retinal angiomatous proliferation; *SRF* subretinal fluid; *IRF* intraretinal fluid; *PED* pigment epithelial detachment; *logMAR* logarithm of minimum angle resolution; *CRT* central retinal thickness; *CT* choroidal thickness.

### Treatment history prior to switching

Patients had received an average of 25.3 ± 21.5 anti-VEGF injections (the mean number of aflibercept injection: 23.6 ± 19.9) with 6.0 ± 5.3 year of the duration before switching. Although they received intensive bimonthly injections of aflibercept, 25 eyes exhibited persistent exudative changes 2 months after the last aflibercept injection (at baseline); therefore, they were switched to faricimab. Duration since the last injection (aflibercept) was 8.1 ± 1.9.

### Effects of faricimab

**Figure 1:**
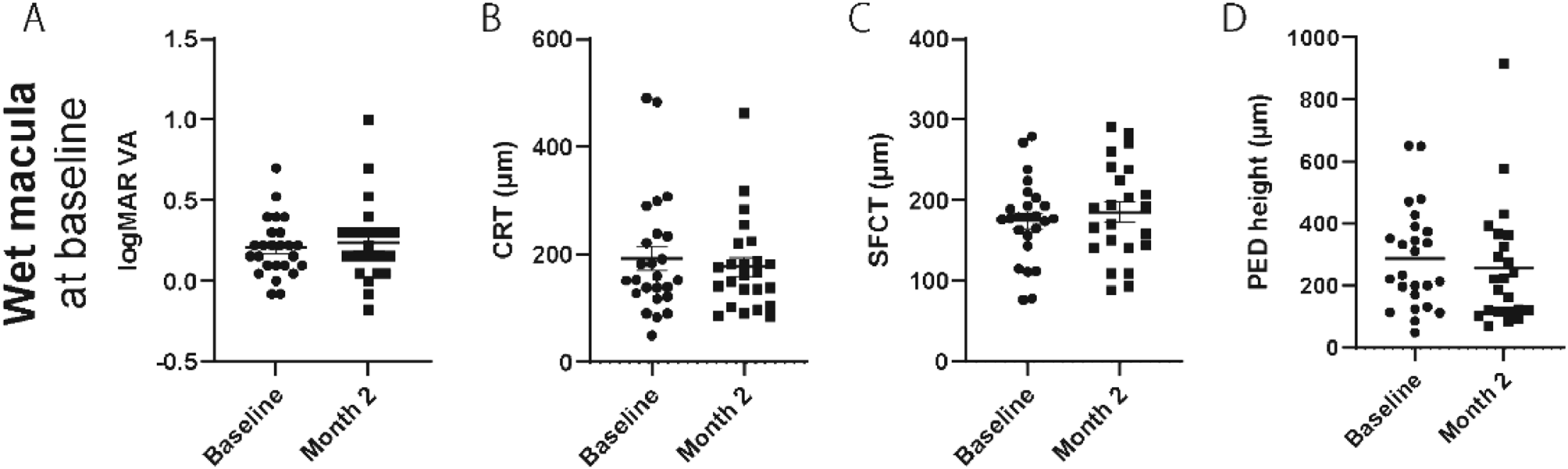
Visual and anatomic outcomes of age-related macular degeneration (AMD) eyes treated with faricimab. Changes in the logarithm of the minimum angle resolution (logMAR) and a comparison between baseline and month 2. (**A**) Visual acuity (VA) (**B**) Central retinal thickness (CRT) (**C**) Subfoveal choroidal thickness (SFCT) (**D**) Maximum pigment epithelial detachment (PED) height.

**Figure 2:**
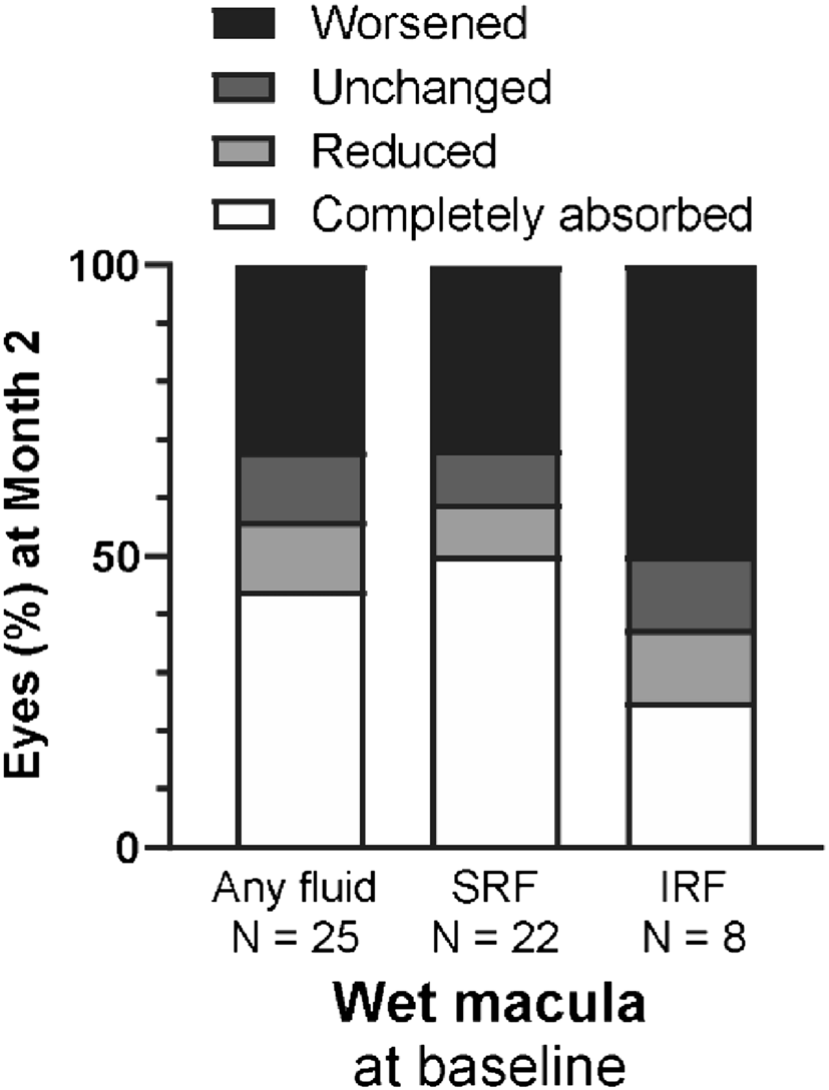
Proportion of eyes with changes (worsened, unchanged, reduced, and completely absorbed) of fluid status. SRF: serous retinal fluid; IRF, intraretinal fluids.

**Figure 3:**
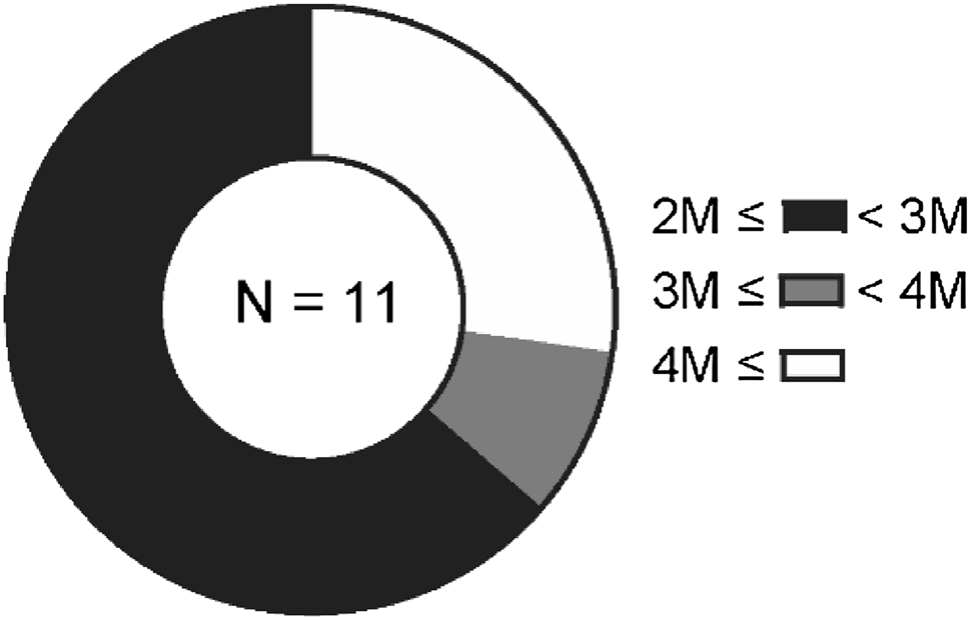
Duration of dry macula after faricimab injection. Pie chart showing how long the retina remained dry beyond 2 months after faricimab injection.

**Figure 4:**
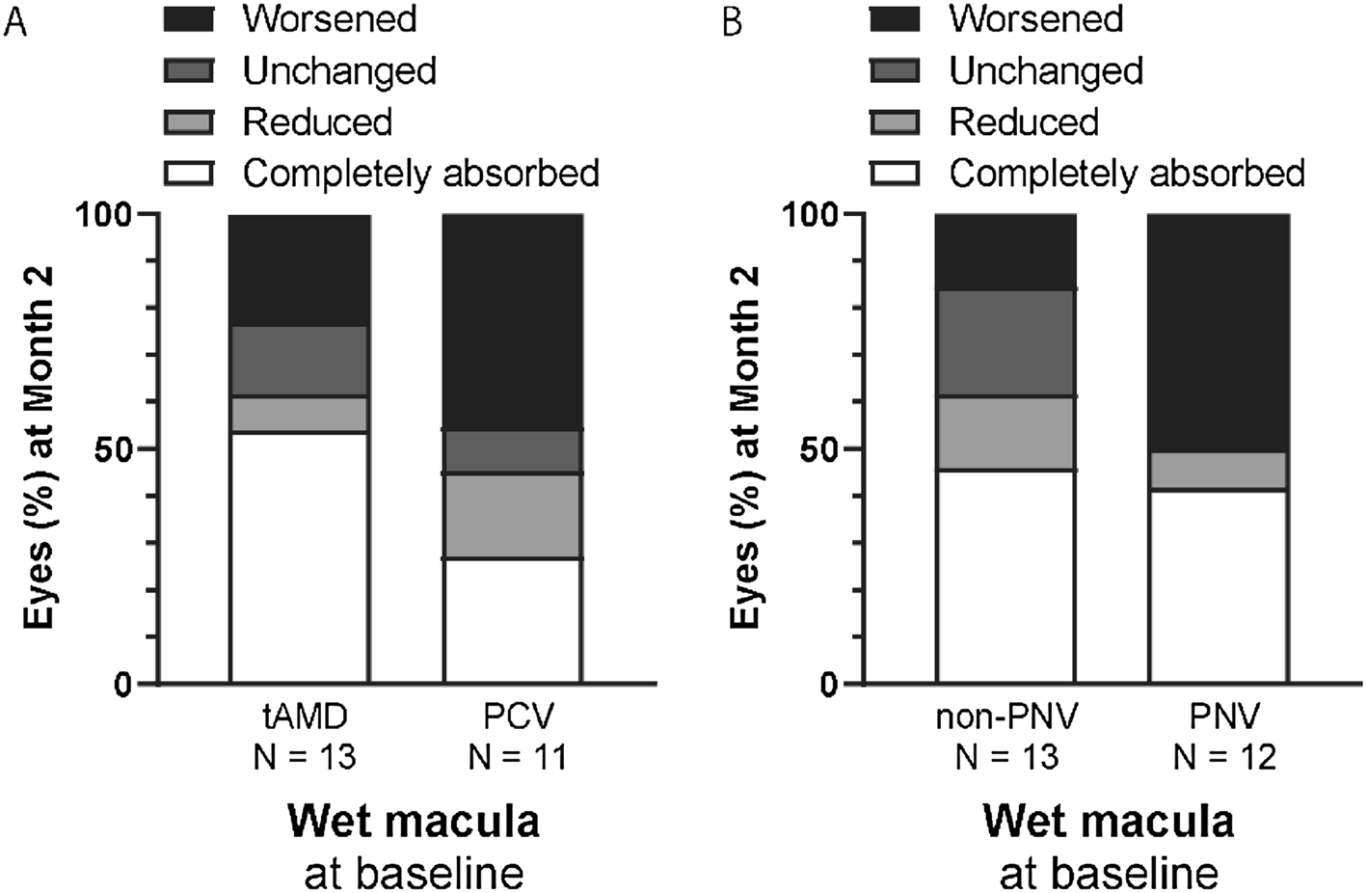
Proportion of eyes with changes (worsened, unchanged, reduced, and completely absorbed) of fluid status. Comparison between tAMD and PCV (**A**), and between non-PNV and PNV (**B**). PCV—polypoidal choroidal vasculopathy; PNV—pachychoroid neovasculopathy; tAMD—typical age-related macular degeneration.

In this group, faricimab injection did not change the logMAR BCVA, CRT, SFCT, or PED height 2 months after faricimab injection (month 2) (Fig. 1). Nonetheless, over 50% of eyes showed reduced or complete remission of fluid. Specific findings on fluid remission and its breakdown by type (SRF and IRF) are illustrated in Fig. 2. Moreover, 27% of the eyes that showed complete fluid remission at month 2 had no fluid recurrence 2 months later (4 months after faricimab injection) (Fig. 3). The effects of treatment conversion were compared between the tAMD and PCV groups (Table 2). There were no significant differences in logMAR BCVA, CRT, SFCT, or PED height between patients with PCV and tAMD at both baseline and month 2 (Supplementary Figure 1). We also investigated whether the pachychoroid phenotypes affected the efficacy of faricimab. As shown in Table 3, those with pachychoroid phenotype were younger than those with non- PNV (*P* = 0.019), and had a thicker choroid (*P* = 0.030). The baseline BCVA was similar between the PNV and non-PNV groups. BCVA significantly worsened in PNV, but not in non-PNV, after a single injection of faricimab (Supplementary Figure 2), although there were no differences in CRT, SFCT, and PED height between baseline and month 2 in both PNV and non-PNV groups. Regarding fluid status at month 2, 53.8% of tAMD eyes showed complete resolution and 23.1% showed worsened status, 27.3% of PCV eyes showed complete resolution and 45.5% showed worsened status, 46.2% of non-PNV eyes showed complete resolution and 15.4% showed worsened status, and 41.7% of PNV eyes showed complete resolution and 50% showed worsened status (Fig. 4).

**Table 2:** Comparison of baseline demographic and clinical characteristics between patients with tAMD and PCV.

	tAMD (n = 13)	PCV (n = 11)	*P* value
Age (years)	76.0 ± 7.7	77.0 ± 9.5	0.788
Sex (male/female)	10/3	9/2	1.000
Axial length (mm)	23.70 ± 1.11	24.30 ± 0.75	0.180
Lens status (phakia/IOL)	5/8	2/9	0.386
Number of previous injections before switching			
Total	18.0 ± 17.0	35.4 ± 22.9	0.054
Aflibercept	17.9 ± 16.7	31.7 ± 21.0	0.101
Ranibizumab	0.0 ± 0.0	2.8 ± 4.6	0.046
Brolucizumab	0.1 ± 0.3	0.6 ± 1.5	0.218
Pegaptanib	0.0 ± 0.0	0.2 ± 0.6	0.287
Duration since the first treatment (years)	4.9 ± 4.9 (1–20)	7.7 ± 5.2 (1–16)	0.210
Duration since the last injection (aflibercept, weeks)	8.2 ± 1.7	7.7 ± 1.8	0.570
Visual acuity (logMAR)	0.24 ± 0.13	0.17 ± 0.22	0.400
Greatest linear dimension (μm)	4264.3 ± 1513.8	5044.4 ± 3146.8	0.472
Fluid			
SRF (+ / −)	10/3	11/0	0.223
IRF (+ / −)	3/10	5/6	0.391
CRT (μm)	181.7 ± 104.8	197.5 ± 113.2	0.738
Subfoveal CT (μm)	167.8 ± 33.9	176.5 ± 61.9	0.678
Maximum PED height (μm)	226.9 ± 187.9	339.9 ± 79.0	0.089

*IOL* intraocular lens; *tAMD* typical age-related macular degeneration; *PCV* polypoidal choroidal vasculopathy; *IRF* intraretinal fluid; *PED* pigment epithelial detachment; *logMAR* logarithm of minimum angle resolution; *CRT* central retinal thickness; *CT* choroidal thickness.

**Table 3:** Comparison of baseline demographic and clinical characteristics between PNV and non-PNV patients.

	Pachychoroid Phenotypes ( +) N = 12	Pachychoroid Phenotypes ( −) N = 13	*P* value
Age (years)	72.8 ± 8.9	81.2 ± 7.2	0.019
Sex (male/female)	8/4	11/2	0.378
Axial length (mm)	23.76 ± 1.15	24.07 ± 0.84	0.475
Lens status (phakia/IOL)	5/7	2/11	0.202
Subtype (tAMD/PCV/RAP)	6/6/0	7/5/1	1.000
Number of previous injections before switching			
Total	26.4 ± 22.8	24.2 ± 20.2	0.810
Aflibercept	25.7 ± 21.9	21.8 ± 17.6	0.641
Ranibizumab	0.1 ± 0.3	2.3 ± 4.4	0.105
Brolucizumab	0.7 ± 1.4	0.0 ± 0.0	0.121
Pegaptanib	0.0 ± 0.0	0.2 ± 0.5	0.347
Duration since the first treatment (years)	6.4 ± 6.1 (0–20)	5.5 ± 4.5 (1–16)	0.637
Duration since the last injection (aflibercept, weeks)	8.1 ± 0.6	8.2 ± 2.5	0.929
Visual acuity (logMAR)	0.16 ± 0.16	0.25 ± 0.18	0.220
Greatest linear dimension (μm)	5156.8 ± 3184.3	4121.8 ± 992.4	0.315
Fluid			
SRF (+ / −)	10/2	12/1	0.593
IRF (+ / −)	5/7	3/10	0.411
CRT (μm)	186.8 ± 108.0	198.7 ± 109.0	0.794
Subfoveal CT (μm)	196.9 ± 37.8	153.7 ± 50.4	0.030
Maximum PED height (μm)	260.4 ± 173.8	311.0 ± 142.4	0.452

*IOL* intraocular lens; *tAMD* typical age-related macular degeneration; *PCV* polypoidal choroidal vasculopathy; *PNV* pachychoroid neovasculopathy; *RAP* retinal angiomatous proliferation; *SRF* subretinal fluid; *IRF* intraretinal fluid; *PED* pigment epithelial detachment; *logMAR* logarithm of minimum angle resolution; *CRT* central retinal thickness; *CT* choroidal thickness.

## Discussion

This study provides a compelling evaluation of the therapeutic potential of faricimab in a cohort of patients with nvAMD refractory to conventional aflibercept anti-VEGF therapy. While the investigation did not discern significant changes in BCVA, CRT, SFCT, or PED height 2 months after faricimab treatment, our findings did reveal a marked reduction or complete remission of fluid in over 50% of patients with aflibercept-refractory nvAMD, indicating a potential therapeutic role for faricimab in this population.

SRF and IRF are signs of MNV activity in both treatment-naïve and treated patients^19^. Persistent or recurrent fluid accumulation during anti-VEGF therapy contributes to vision loss over time^20,21^. In our study, complete fluid remission was observed after a single faricimab injection in 50% of patients with persistent SRF. Importantly, 27% of the patients who demonstrated complete fluid remission at month 2 displayed no fluid recurrence at 4 months, suggesting that faricimab may offer a durable treatment response in some patients with refractory nvAMD. This notable fluid reduction, even without significant changes in other clinical markers, could potentially contribute to the long-term preservation of vision and improved quality of life in patients. In contrast to SRF, only 25% of patients with IRF showed complete fluid remission. Previous reports have indicated that the presence of IRF during treatment is strongly associated with worse visual outcomes^21,22^ and higher rates of atrophy and fibrosis^23^. For eyes with persistent IRF, the effect of switching to faricimab is limited. Thus, intensive treatment using other treatment options, such as other anti-VEGF reagents and/or photodynamic therapy, may be needed for better visual outcomes.

In an investigation on the efficacy of faricimab across different nvAMD subtypes, the rate of fluid resolution was lower in PCV than in tAMD, although no significant differences were observed between tAMD and PCV in terms of BCVA, CRT, SFCT, or PED height. Moreover, our analysis of the pachychoroid phenotype showed that younger patients with thicker choroids exhibited worsening of BCVA following a single faricimab injection, accompanied by a higher rate of worsened fluid status, highlighting that specific phenotypic factors, such as pachychoroid and/or polypoidal lesions, may influence the response to faricimab treatment.

This study has several limitations. This includes a short follow-up period and a small sample size. In particular, there was only one case of RAP, making it difficult to analyze RAP cases. Additional studies, ideally conducted over a longer period and employing larger sample sizes, are required to validate our results. Regarding fluid analysis, our study evaluated the existence of SRF and IRF, owing to the lack of quantification software. Further studies with quantification of the area and volume of fluid could provide a more accurate judgement of the treatment effectiveness for SRF and IRF^24^.

In conclusion, our findings suggest that faricimab, a dual-action anti-VEGF and anti-Ang-2 agent, may be a viable treatment alternative for aflibercept-refractory nvAMD, particularly for patients exhibiting persistent exudative changes. However, patient-specific factors and the nvAMD subtype may influence the treatment response, emphasizing the importance of personalized medicine approaches in the management of this complex condition. Further research including randomized controlled trials with larger patient cohorts and longer follow-up periods is required to confirm and expand upon our findings.

### Supplementary Information

Supplementary Information.

## Data Availability

The datasets analyzed in the current study are available from the corresponding author upon request.
