# Supplementary material for: Therapeutic effects of faricimab on aflibercept-refractory age-related macular degeneration
Source: Sci Rep. 2023 Nov 30;13:21128. doi: 10.1038/s41598-023-48190-6 (PMC10689783; doi:10.1038/s41598-023-48190-6)
Supplement: Supplementary file 1 — Supplementary Information. [file 41598_2023_48190_MOESM1_ESM.docx]

**Supplementary Figure 1.**

**
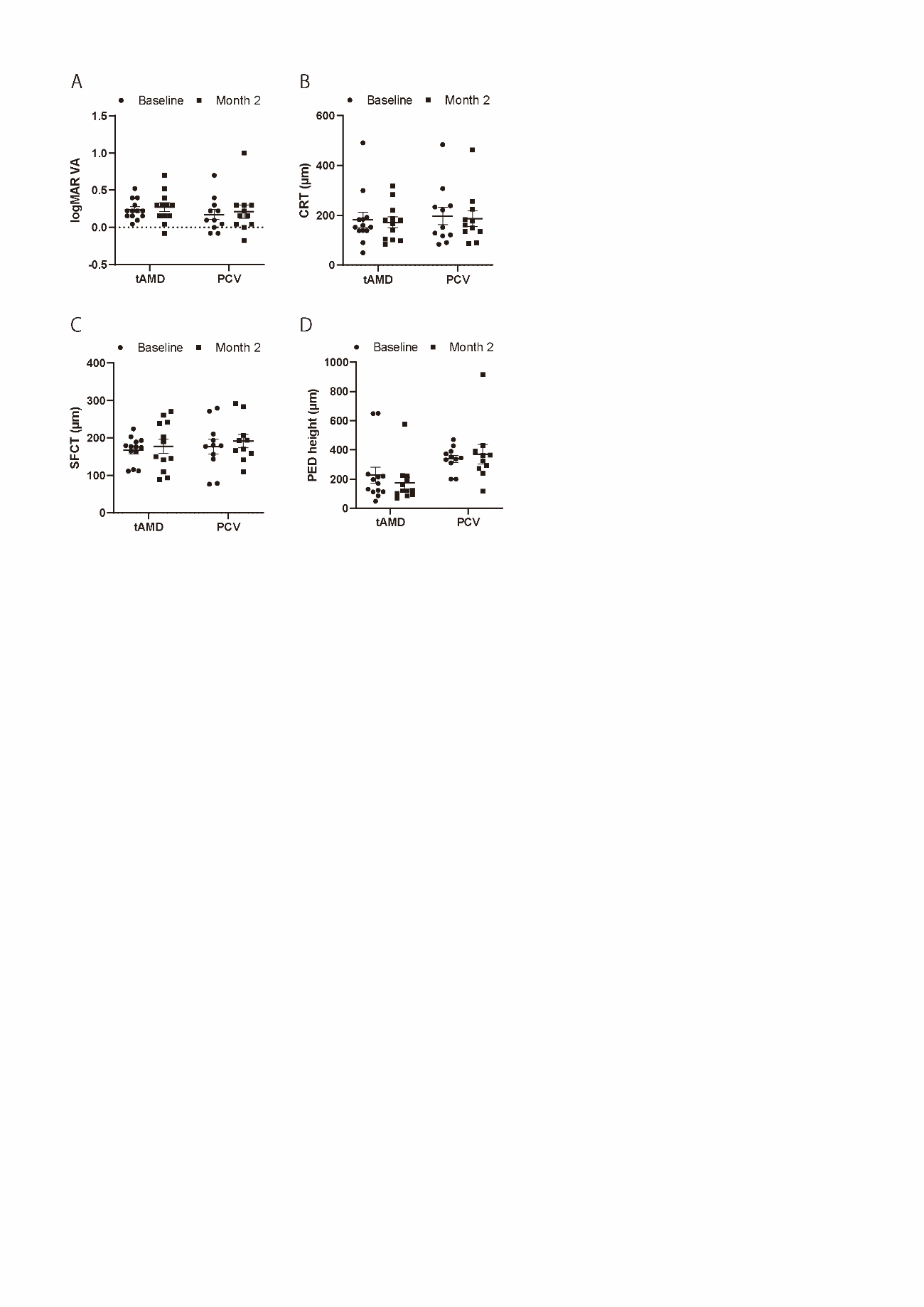
**

**Supplementary Figure 1. Visual and anatomic outcomes of typical AMD and PCV eyes treated with faricimab.**

Changes in the logarithm of the minimum angle resolution (logMAR) and a comparison between baseline and month 2.

(**A**) Visual acuity (VA)

(**B**) Central retinal thickness (CRT)

(**C**) Subfoveal choroidal thickness (SFCT)

(**D**) Maximum pigment epithelial detachment (PED) height

PCV, polypoidal choroidal vasculopathy; tAMD, typical age-related macular degeneration.

**
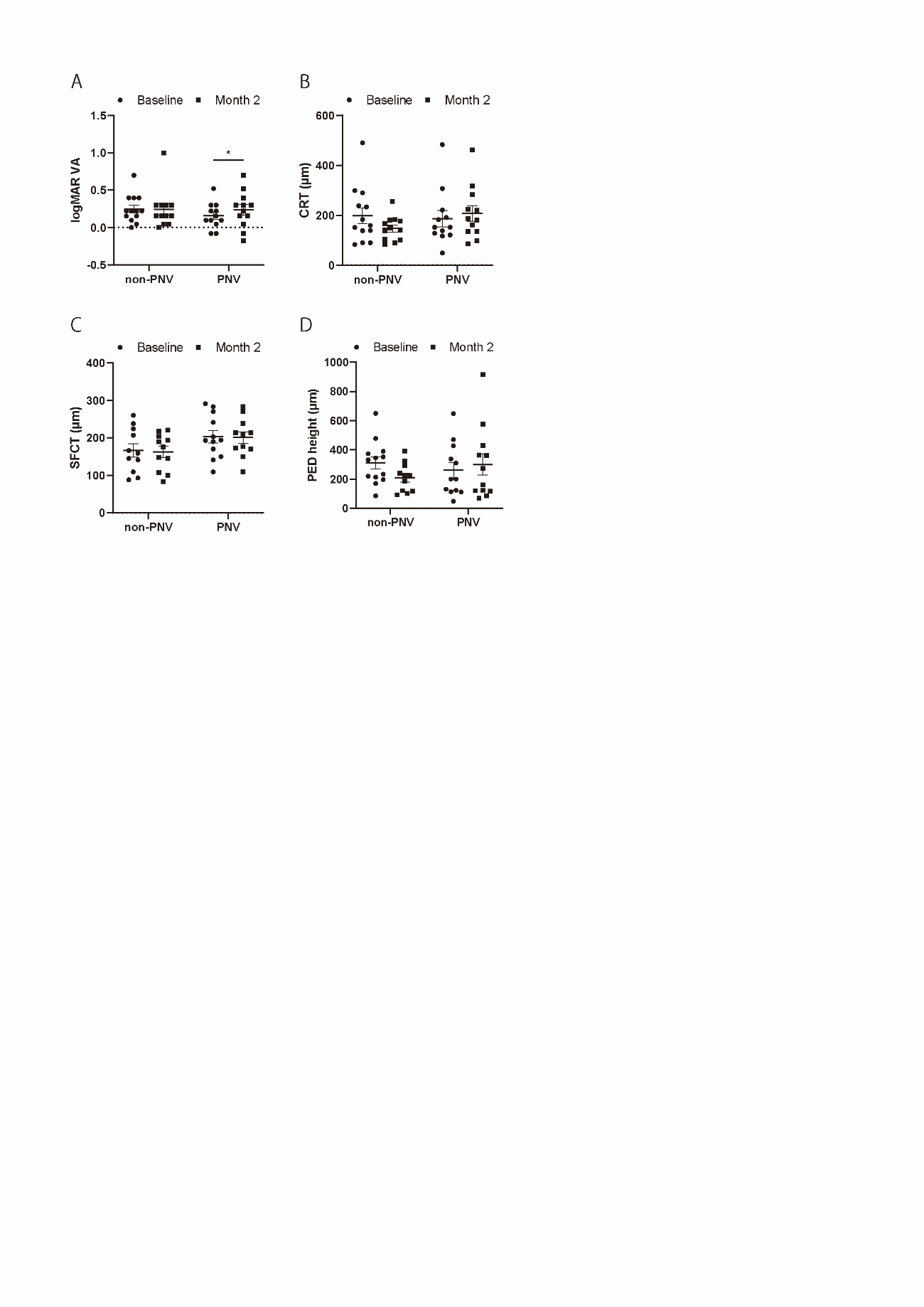
Supplementary Figure 2.**

**Supplementary Figure 2. Visual and anatomic outcomes of non-PNV and PNV eyes treated with faricimab.**

Changes in the logarithm of the minimum angle resolution (logMAR) and a comparison between baseline and month 2.

(**A**) Visual acuity (VA)

(**B**) Central retinal thickness (CRT)

(**C**) Subfoveal choroidal thickness (SFCT)

(**D**) Maximum pigment epithelial detachment (PED) height

PCV, polypoidal choroidal vasculopathy; tAMD, typical age-related macular degeneration.
